# Supplementary material for: A wireless controlled robotic insect with ultrafast untethered running speeds
Source: Nat Commun. 2024 May 8;15:3815. doi: 10.1038/s41467-024-47812-5 (PMC11078929; doi:10.1038/s41467-024-47812-5)
Supplement: Supplementary file 1 — Supplementary Information [file 41467_2024_47812_MOESM1_ESM.pdf]

Supplementary Information

A Wireless Controlled Robotic Insect with Ultrafast Untethered Running Speeds

Zhiwei Liu[1,2,3,4†], Wencheng Zhan[5†], Xinyi Liu[1], Yangsheng Zhu[1], Mingjing Qi[1,2,3,4], Jiaming Leng[1,2,3,4], Lizhao Wei[1], Shousheng Han[6], Xiaoming Wu[6]*, Xiaojun Yan[1,2,3,4]*
*Corresponding authors:
Xiaoming Wu (imewuxm@tsinghua.edu.cn), Xiaojun Yan (yanxiaojun@buaa.edu.cn)
†These authors contributed equally to this work.

Supplementary Information contains:

Supplementary Notes 1-10
Supplementary Figures 1-9
Supplementary Tables 1-13
Supplementary References

Supplementary Notes 1-10

S1. Modeling of Electromagnetic Actuator

The electromagnetic force applied on the permanent magnet determines the performance of the electromagnetic actuator and the BHMbot. Therefore, it is necessary to establish the mathematical model of the electromagnetic force.

Supplementary Fig. 1a shows the schematic of the electromagnetic actuator composed of a permanent magnet and a hollow coil (the elastic component is hidden). Since the wire diameter of the coil is far less than its inner diameter, it is simplified as a cylindrical shell with a diameter of r and a length of l, ignoring the thickness. The permanent magnet has a diameter of R and a length of L. Surface 1 and 2 are the upper and lower surfaces of the permanent magnet respectively. It is assumed that the magnetic field generated by the coil is uniformly distributed along the radial direction, ignoring the influence of the coil self-inductance.

The electromagnetic force applied on the permanent magnet is related to the strength of the magnetic field, so it is necessary to calculate the distribution of the magnetic field along the axis generated by the energized solenoid. As shown in Supplementary Fig. 1b, according to the Bio-Savar law of static magnetism, the magnetic induction intensity generated by the current element idl on the axis is:

$$d\mathbf{B} = \frac{\mu_0 i}{4\pi} \frac{\mathbf{d} \times \mathbf{r_1}}{|r'|^3} \tag{1}$$

where μ_0 is the permeability of vacuum ($4\pi \times 10^{-7}$ Wb Agm^{-1}). The axial component of the magnetic field intensity generated by the whole coil is the integration of the above formulas along the circumferential and axial directions.

$$B_z = \int_0^l \int_0^{2\pi} \frac{\mu_0 N I r^2}{4\pi (r^2 + z^2)^{3/2}} d\psi dz \tag{2}$$

Therefore, the final expression of the B_z is:

$$B_z(z)=\frac{\mu_0 NI}{2l}\left(\frac{l-z}{\sqrt{(l-z)^2+r^2}}+\frac{z}{\sqrt{z^2+r^2}}\right) \tag{3}$$

The electromagnetic force exerted on the magnet can be given as[1]:

$$F_z=\int(\mathbf{M}_{\mathbf{mag}}\cdot\hat{\mathbf{n}})B_z dS=\frac{1}{\mu_0}\int(B_r\hat{\mathbf{z}}\cdot\hat{\mathbf{n}})B_z dS \tag{4}$$

where M_{mag} is the intensity of magnetization that is assumed to be invariable along the axial direction of the coil. Therefore, the electromagnetic force is:

$$F_{EM}=\frac{B_r(B_{z1}-B_{z2})A}{\mu_0}=\frac{\pi B_r(B_{z1}-B_{z2})R^2}{\mu_0} \tag{5}$$

$$\begin{aligned} B_{z1}&=B_z(z)=\frac{\mu_0 NI}{2l}\left(\frac{l-z}{\sqrt{(l-z)^2+r^2}}+\frac{z}{\sqrt{z^2+r^2}}\right)\\ B_{z2}&=B_z(z+L)=\frac{\mu_0 NI}{2l}\left(-\frac{z+L-l}{\sqrt{(z+L-l)^2+r^2}}+\frac{z+L}{\sqrt{(z+L)^2+r^2}}\right) \end{aligned} \tag{6}$$

where B_r is the residual magnetization intensity of the magnet (1.3 T); B_{z1} and B_{z2} are the magnetic field intensity of surface 1 and 2 of the magnet respectively; R is the radius of the magnet; r is the radius of the hollow coil, as shown in Supplementary Fig. 1a.

S2. Optimization of Electromagnetic Actuator

Supplementary Equation (5) and (6) indicate that the influencing factors of the electromagnetic force F_{EM} include the amplitude of the working current I, the turns of the coil N, the relative distance between the magnet and the coil z, the geometric dimensions of the coil (r, l, R, and L). It is unnecessary to optimize the first two factors because of the simple linear relationship between them and F_{EM}. Therefore, we mainly discuss the optimization of the relative distance z and the geometric dimensions (r, l, R, and L).

Supplementary Fig. 1c shows the variation curve of F_{EM} versus the relative distance z. Since the magnetic field generated by the coil is symmetric with respect to the central plane of the coil, the variation curve is also centrosymmetric relative to the point O ($z = 0.5l - 0.5L$), at which the central planes of the magnet and the coil are overlapped. At two positions equidistant from the point O, the electromagnetic force is equal in

magnitude but opposite in direction. Therefore, we only consider the situation on one side of point O in the following discussions. Especially, there is a peak point P on the variation curve, where F_{EM} reaches the maximum. The relative distance z corresponding to the peak point is defined as z_{op}.

Supplementary Equation (5) indicates that F_{EM} is proportional to the difference of magnetic field intensity between the two surfaces of the magnet. Supplementary Fig. 1d shows the variation of the magnetic field intensity generated by the coil along the axis B_z. The z_{op} can be determined by identifying the two points on the axis of the coil separated by a distance of L with the maximum difference of B_z. Therefore, we first identify the point with the maximum value of the derivative of B_z. The expression of the derivative is as follows:

$$\begin{aligned} f_{\Delta B_z}' &= \left[\frac{\mu_0 NI}{2} \left(\frac{l-x}{\sqrt{(l-x)^2+r^2}} + \frac{x}{\sqrt{x^2+r^2}} \right) \right]' \\ &= \frac{\mu_0 NI}{2} r^2 \left(-\frac{1}{\left[(l-x)^2+r^2\right]^{3/2}} + \frac{1}{\left[x^2+r^2\right]^{3/2}} \right) \end{aligned} \tag{7}$$

When $x = l$ (point D in Supplementary Fig. 1d), the derivative reaches the maximum value, indicating the maximum gradient of B_z. Therefore, it can be assumed that when the length of the permanent magnet L is far smaller than the length of the coil l, the maximum electromagnetic force is achieved when the center of the magnet is positioned at the bottom surface of the coil (i.e., $z = l - L/2$), which corresponds to the previous peak point P. It should be noted that the gradient of B_z near point D is not symmetric, and the gradient on the right side (i.e., away from the coil) decreases more gradually. Therefore, the optimal distance z_{op} is often slightly larger than $l - L/2$. Supplementary Table 2 compares the theoretical and estimated distance z_{op} for magnets with different lengths. As the length of the magnet decreases, the relative error becomes smaller. When the length of the magnet is equal to the length of the coil, the relative error reaches the maximum but is still within an acceptable range of 11.5%. Therefore, it is feasible to use the estimated value ($l < L$). When the length of the magnet is greater than the length of the coil, the above conclusion is no longer applicable.

In addition to the relative distance z, the geometric parameters of the magnet and the coil also have significant influences on F_{EM}. Based on Supplementary Equation (5), the electromagnetic force is proportional to the square of the radius R of the magnet. Therefore, when the diameter of the coil is fixed, a larger diameter of the magnet is preferred. However, it is important to ensure that the magnet can move smoothly within the coil without resistance. Considering the above requirement and possible error of manual assembly, the diameter of the coil is set to be 0.3 mm larger than the diameter of the magnet. Under this constraint, the variation curve of F_{EM} versus the radius R is shown as Supplementary Fig. 1e, which indicates that F_{EM} rises gradually as the radius R increases, with the gradient of the variation curve decreasing. Considering that an increase of the radius R leads to a rapid increase in mass, the radius R of the magnet is set to be below 2 mm ($r < 2.15$ mm) because of the low gradient of the curve after $R > 2$ mm ($r > 2.15$ mm).

Supplementary Fig. 1f shows the variation of the electromagnetic force versus two length parameters l and L. In the calculations, the magnet is always maintained at the optimal position. It can be observed that the variation trends of the two curves are similar. As the length increases, the electromagnetic force initially increases rapidly and then gradually levels off. Since an increase in length also leads to an increase in the mass of the actuator, the length of the actuator should be within the range of rapid increase (within 3mm) to achieve higher output power.

The factors determining the electromagnetic force are diverse and interrelated. Therefore, the optimization is essentially a problem of solving the extremum of a multivariate function. We utilize an embedded function named "fmincon" of MATLAB to solve the optimization problem. Considering the mass of the actuator must be considered during the optimization process, we select the force efficiency as the objective function for optimization, which is defined as the ratio of the maximum electromagnetic force to the weight of the actuator. The variables include the relative distance z and the dimensional parameters (R, L, r, l) of the actuator. The model is formulated as follows:

$$\begin{aligned}\text{fun} &= f(R,L,r,l,z)\\ &= -\frac{F_{\text{EM}}}{m_{\text{act}}g} = -\frac{F_{\text{EM}}}{(\rho_{\text{mag}}\pi R^2 L + N\cdot 2\pi r\cdot \rho_{\text{wing}}\pi {r_{\text{wing}}}^2)g}\end{aligned} \tag{8}$$

with

$$s.t.\begin{cases} r-R \geq 0.15\text{mm} \\ l-L \geq 0.5\text{mm} \\ 0 < R \leq 2\text{mm}, 0 < r \leq 2.15\text{mm} \\ 0 < L \leq 3\text{mm}, 0 < l \leq 3\text{mm} \end{cases} \tag{9}$$

where m_{act} is the mass of the actuator; ρ_{mag} is the density of the magnet; ρ_{wing} is the density of the copper wire of the coil; r_{wing} is the radius of the copper wire of the coil.

The optimized and revised results are shown in Supplementary Table 3. The force efficiency of the optimized actuator rises to 21.8, which demonstrates an enhancement of 20.8% compared to the initial actuator (16.6).

S3. Design of the transmission mechanism

The design of the transmission is inspired by a four-bar linkage transmission used in micro flapping vehicles[2]. As shown in Fig. 1c, the planar four-bar linkage transmission acts as a joint connecting the actuator and the front leg, which is composed of several linkages and flexible hinges. One end of the transmission (L3) is connected to the magnet and the other (L1) is connected to the front leg. Then, the reciprocating motion of the magnet can be transformed into the swing motion of the front leg through the transmission. There are two key parameters for the transmission: the length of the linkage L1 (l_{L1}) and the thickness of the hinges (t_{k}). l_1 determines the transmission ratio[3] and t_{k} determines the transmission rigidity:

$$\lambda \approx \frac{1}{l_{\text{L1}}} \tag{10}$$

$$k_{\text{t}} = \frac{E_{\text{k}} t_{\text{k}}^3 w_{\text{k}}}{12 l_{\text{k}}} \tag{11}$$

where λ is the transmission ratio of the transmission; k_{t} is the stiffness coefficient of the transmission; E_{k}, t_{k}, w_{k} and l_{k} are the elasticity modulus, thickness, width and length of the hinge. Based on the performance of

the electromagnetic actuator (output force and displacement), we can obtain the initial design values of l_{L1} and t_k.

S4. Scaling design of the BHMbot

To investigate the influence of the size on the performance of the BHMbot, we designed a series of prototypes with different body lengths ranging from 10 to 25 mm at an interval of 5 m (prototype #2.1-2.4 with lengths of 10, 15, 20 and 25 mm respectively). The ratio of the body lengths of two prototypes is selected as the scaling parameter (ϕ = BL_2/BL_1). Considering the limitation of the materials, the thicknesses of the supporting frames, cantilevers, and rear legs remain constant for all prototypes (0.15 mm), which are cut from sheets composed of three orthogonal 50-μm-thick carbon fiber layers. Other geometrical parameters of every component are determined by scaling calculation based on the parameters of prototype #2.2 (15 mm). Part of the key parameters are listed in Supplementary Table 4. It is noted that the magnet and hollow coil of the actuator are both standardized products, which means that the scaling of these two components is also limited. Therefore, we tend to select the products with the closest parameters compared to the design values. Furthermore, the transmission and front leg of the BHMbot is a laminated structure, which is composed of two carbon fiber layers, two adhesive layers and a polyimide film layer (hinge). The thicknesses of the carbon fiber layers and adhesive layers are constant during the scaling process. Considering that the thickness of the hinge determines the rigidity of the transmission (see Supplementary Note 3), the parameter is also scaled (Supplementary Table 4).

S5. A simplified planar model of the BHMbot

Based on the experimental results, the mass and position of the payloads both have a significant influence on the locomotion performance of the BHMbot. A simplified planar dynamic model is developed to investigate the dynamic characteristics of the BHMbot with payloads, which consists of two rigid bodies M_b (including the support frames, actuators, transmissions, and rear legs) and M_f (front legs), a lumped masses

M_p (payloads), as shown in Fig. 2e and 2f. To focus on the effect of the payloads, several simplifications are made:

(1) The output parameters of the two electromagnetic actuators are the same. Therefore, two front legs connecting with two actuators respectively are simplified to one front leg M_f.

(2) The pin joint between two rigid bodies M_b and M_f is simplified as a torsional spring-damper (k_θ-d_θ) with an elastic torque (T_k) and a damping torque (T_d).

(3) The action of the electromagnetic actuator and transmission is simplified as a sinusoidally varying torque applied on M_f (T_a).

(4) The contact between the legs and the ground is simplified as a vertical spring-damper (k-d) with a normal force in the vertical direction (F_N) and a friction force in the lateral direction (F_f).

We utilize the Lagrangian method to establish the dynamical equations of the BHMbot. Four generalized coordinates q_i (i = 1,2,3,4) are selected to characterize the locomotion status of the BHMbot: x_{Mb}, y_{Mb}, θ_b, and θ_f. x_{Mb} and y_{Mb} are the coordinates of the COM of M_b in the inertial coordinate system (x-o-y). θ_b is the body tilt angle relative to the horizontal axis of the inertial coordinate system. θ_f is the swing angle of the front leg relative to the vertical axis of the body coordinate system (x'-o-y'). The Lagrange function can be given as:

$$L = E_k - E_p \tag{12}$$

$$E_k = 0.5m_b(\dot{x}_{Mb}^2 + \dot{y}_{Mb}^2) + 0.5m_p(\dot{x}_{Mp}^2 + \dot{y}_{Mp}^2) + 0.5m_f(\dot{x}_{Mf}^2 + \dot{y}_{Mf}^2) + 0.5J_b\dot{\theta}_b^2 + 0.5J_f\dot{\theta}_f^2 \tag{13}$$

$$\begin{aligned} E_p = & m_b g y_{Mb} + m_p g(y_{Mb} + 0.5h\cos\theta_b + (l_3 - l_1)\sin\theta_b) \\ & + m_f g(y_{Mb} + l_2\sin\theta_b - 0.5l_f\cos(\theta_b + \theta_f)) + 0.5k_\theta\theta_f^2 \end{aligned} \tag{14}$$

where x_{Mp} and y_{Mp} are the coordinates of M_p in the inertial coordinate system (x-o-y); x_{Mf} and y_{Mf} are the coordinates of the COM of M_f in the inertial coordinate system (x-o-y); m_b, m_p and m_f are the masses of M_b, M_p and M_f; J_b and J_f are the moments of inertia of M_b and M_f; h is the height of M_b; l_1 is the relative distance between the COM of M_b and the rear end of the body (Fig. 2f); l_2 is the relative distance between the COM of M_b and the front end of the body (Fig. 2f); l_3 is the relative distance between M_p and the rear end of the

body (Fig. 2f); l_f is the length of the front legs (Fig. 2f); k_θ is the torsional elastic coefficient of the torsional spring-damper (Fig. 2e).

Based on the Lagrange equations in Supplementary Equation (15), the dynamic equations of the BHMbot can be expressed as Supplementary Equation (16) and (17).

$$\frac{d}{dt}\frac{\partial L}{\partial \dot{q}_i}-\frac{\partial L}{\partial q_i}=Q_i \ \left(\text{i=1,2,3,4}\right) \tag{15}$$

$$\begin{bmatrix} m_\mathrm{b}+m_\mathrm{p}+m_\mathrm{f} & 0 & 0 & 0 \\ 0 & m_\mathrm{b}+m_\mathrm{p}+m_\mathrm{f} & 0 & 0 \\ 0 & 0 & J_\mathrm{b}+J_\mathrm{f} & J_\mathrm{f} \\ 0 & 0 & J_\mathrm{f} & J_\mathrm{f} \end{bmatrix}\begin{bmatrix} \ddot{x}_\mathrm{Mb} \\ \ddot{y}_\mathrm{Mb} \\ \ddot{\theta}_\mathrm{b} \\ \ddot{\theta}_\mathrm{f} \end{bmatrix}=\begin{bmatrix} Q_1 \\ Q_2-\left(m_\mathrm{b}+m_\mathrm{p}+m_\mathrm{f}\right)g \\ Q_3-m_\mathrm{f}gl_2\cos\theta_\mathrm{b}-0.5m_\mathrm{f}gl_\mathrm{f}\sin\left(\theta_\mathrm{b}+\theta_\mathrm{f}\right) \\ Q_4-0.5m_\mathrm{f}gl_\mathrm{f}\sin\left(\theta_\mathrm{b}+\theta_\mathrm{f}\right)-k_\theta\theta_\mathrm{f} \end{bmatrix} \tag{16}$$

$$\begin{bmatrix} Q_1 \\ Q_2 \\ Q_3 \\ Q_4 \end{bmatrix}=\begin{bmatrix} F_\mathrm{fr}+F_\mathrm{ff}+F_\mathrm{dx} \\ F_\mathrm{Nr}+F_\mathrm{Nf}+F_\mathrm{dy} \\ \left(l_1\sin\theta_\mathrm{b}+l_\mathrm{r}\cos\theta_\mathrm{b}\right)F_\mathrm{fr}+\left(-l_1\cos\theta_\mathrm{b}+l_\mathrm{r}\sin\theta_\mathrm{b}\right)F_\mathrm{Nr}+\left(-l_2\sin\theta_\mathrm{b}+l_\mathrm{f}\cos\left(\theta_\mathrm{b}+\theta_\mathrm{f}\right)\right)F_\mathrm{ff}+\left(l_2\cos\theta_\mathrm{b}+l_\mathrm{f}\sin\left(\theta_\mathrm{b}+\theta_\mathrm{f}\right)\right)F_\mathrm{Nf} \\ l_\mathrm{f}\cos\left(\theta_\mathrm{b}+\theta_\mathrm{f}\right)F_\mathrm{ff}+l_\mathrm{f}\sin\left(\theta_\mathrm{b}+\theta_\mathrm{f}\right)F_\mathrm{Nf}+T_\mathrm{a}+T_\mathrm{d} \end{bmatrix} \tag{17}$$

where F_fr, F_ff are the frictional forces applying on the rear and front legs respectively; F_Nr, F_Nf are the normal forces applying on the rear and front legs respectively; F_dx, F_dy are the air damping forces applying on the rigid body M_b in the x-axis and y-axis direction respectively; T_d is the damping torque generated by the air; T_a is the sinusoidally varying torque of the actuators:

$$T_\mathrm{a}=T_0\sin(2\pi\omega t) \tag{18}$$

Based on the spring-damper model between the feet and the ground, the normal forces are given as (19) and (20) when the feet are in contact with the ground. If one foot is off the ground, the corresponding normal force and frictional force are both equal to zero.

$$\begin{cases} F_\mathrm{Nr}=sign(-y_\mathrm{fr})\times(-ky_\mathrm{fr}-d\dot{y}_\mathrm{fr}) \\ F_\mathrm{Nf}=sign(-y_\mathrm{ff})\times(-ky_\mathrm{ff}-d\dot{y}_\mathrm{ff}) \end{cases} \tag{19}$$

$$\begin{cases} F_\mathrm{fr}=sign(-\dot{x}_\mathrm{fr})\mu F_\mathrm{Nr} \\ F_\mathrm{ff}=sign(-\dot{x}_\mathrm{ff})\mu F_\mathrm{Nf} \end{cases} \tag{20}$$

where x_r, y_r are the coordinates of the rear foot in the inertial coordinate system; x_f, y_f are the coordinates of the front foot in the inertial coordinate system; k, d are the elastic coefficient and damping coefficient

respectively when the feet are in contact with the ground; μ is the friction coefficient between the feet and ground.

The air damping forces in (17) of the rigid body M_b are expressed as:

$$F_{dx} = 0.5 C_D \rho_{air} \dot{x}_{Mb} \left| \dot{x}_{Mb} \right| A_{bx} \tag{21}$$

$$F_{dy} = 0.5 C_D \rho_{air} \dot{y}_{Mb} \left| \dot{y}_{Mb} \right| A_{by} \tag{22}$$

where C_D is the air damping coefficient; ρ_{air} is the density of air; A_{bx} is the area of the surface perpendicular to the *x-axis* for the body M_b; A_{by} is the area of the surface perpendicular to the *y-axis* for the body M_b.

The simulation parameters used in the modeling are listed in Supplementary Table 6. Supplementary Fig. 3 shows the simulation results of the generalized coordinates used in the model when the driving frequency is 200 Hz.

S6. Optimization parameters analysis

To enhance the locomotion performance of the BHMbot with payloads, several structural parameters related to the locomotion performance are selected to be optimized based on the planar dynamical model (Fig. 2e and 2f), including the initial relative distance between the hollow coil and the magnet z, the width of the cantilever of the electromagnetic actuator w_c, the initial body tilt angle θ_0, and the length of rear legs l_r.

The initial relative distance between the hollow coil and the magnet z is a key structural parameter that influences the electromagnetic force (Supplementary Fig. 1c) and the active torque T_a. The relation between the active torque T_a used in the planar model and the output force of the electromagnetic actuators F_{EM} is:

$$T_a = \frac{F_{EM}}{\lambda} \tag{23}$$

where λ is the transmission ratio of the planar four-bar linkage mechanism of BHMbot. F_{EM} can be calculated via Supplementary Equation (5) and (6).

w_c is a key parameter determining k_θ used in the planar model, which represents the sum of the stiffness coefficients of the transmission mechanism and the cantilever of the electromagnetic actuators:

$$\begin{cases} k_\theta = k_t + \dfrac{k_c l_c}{\lambda} \\ k_t = \dfrac{E_k t_k^3 w_k}{12 l_k} \\ k_c = \dfrac{3E_c I_c}{l_c^3} = \dfrac{E_c w_c t_c^3}{4 l_c^3} \end{cases} \tag{24}$$

where k_t is the stiffness coefficient of the transmission mechanism; k_c is the stiffness coefficient of the cantilever; λ is the transmission ratio of the transmission; E_k, t_k, w_k and l_k are the elasticity modulus, thickness, width and length of the flexible hinge of the transmission mechanism; E_c, I_c, l_c, w_c and t_c are the elasticity modulus, inertia moment, length, width and thickness of the cantilever.

Based on the Section "Design and moving mechanism", initial body tilt angle θ_b is essential for generating the forward friction force or momentum required for locomotion, which only depends on the geometric parameters of the BHMbot:

$$\theta_b = \arctan\left(\frac{l_f - l_r}{\mathrm{BL}}\right) \tag{25}$$

where l_f is the length of the front leg; l_r is the length of the rear leg; BL is the length of the body (15 mm for prototype #2.2).

The above equations establish the correlation between the four structural parameters to be optimized and the planar dynamical model.

S7. Influencing factors of the optimal payload mass m_{op}

Based on the above dynamical model, we can obtain simulation values of the running speed of the BHMbot under varying payload mass. The experimental results reveal the presence of an optimal payload mass (denoted as m_{op}), which corresponds to the maximum running speed V_{max}. To investigate the influencing factors of m_{op}, we select four key parameters for simulation analysis, including the relative location of the COM of the payload mass l_3, the amplitude of the active torque T_0, the initial body tilt angle of the rigid body θ_0, and the torsional stiffness of the actuation system k_θ, as shown in Fig. 3h and Supplementary Fig. 4. Fig.

3h shows that the initial tilt angle θ_0 has an influence on m_{op}. As θ_0 increases, m_{op} also increases with a slight decrease in v_{max}. Consequently, there is an optimal θ_0 for a given payload mass, assuming the other parameters remain constant. Supplementary Fig. 4a shows that an increase in l_3 results in a decrease in m_{op}. Nevertheless, it should be noted that a larger l_3 also results in a higher running speed across the entire range of payload mass. It indicates that the payload should be positioned towards the front of the BHMbot to achieve a faster running speed. Supplementary Fig. 4b shows that both m_{op} and V_{max} increase with the increase of T_0, which is determined by the output force of the electromagnetic actuator. Supplementary Fig. 4c shows that m_{op} decreases slightly with an increase in k_θ. In summary, m_{op} is a function determined by the above parameters.

S8. Detailed discussion on the scaling effects of the BHMbot

In the scaling analysis, it is assumed that all the geometric parameters of the components are scaled down based on the ratio of the body length ϕ (BL_2/BL_1). Thus, the total mass of the BHMbot will scale down cubically with size decreasing ($m_2/m_1 = \phi^3$). The torsional stiffness of the front leg can be calculated by Supplementary Equation (24), and it also scales down cubically with the size decreasing ($k_{\theta 2}/k_{\theta 1} = \phi^3$). The resonant frequency of the BHMbot is determined by the dynamic characteristics of the whole robot. It is difficult to obtain an analytical expression of the resonant frequency. Therefore, we select the resonant frequency of the actuation system (including the actuator, transmission and the front leg) as an estimation:

$$\begin{cases} f_r = \dfrac{\sqrt{k_\theta / I_f}}{2\pi} \\ I_f = \dfrac{1}{3} m_f l_f^2 \propto \phi^5 \end{cases} \tag{26}$$

where I_f, m_f and l_f are the moment of inertia, mass and length of the front leg respectively. Thus, the scaling of the resonant frequency is $f_{r2}/f_{r1} = \phi^{-1}$. The electromagnetic force generated by the actuator can be calculated by Supplementary Equation (5) and (6). It is assumed that the current amplitude and wire diameter of the coil remain constant (the number of turns of the coil is scaled). Therefore, the magnetic field strengths B_{z1} and B_{z2}

are not affected by the scaling process, which means that the scaling of the electromagnetic force is $F_{EM2}/F_{EM1} = \phi^2$. The torque applied on the front leg is calculated by Supplementary Equation (23), and the scaling is $T_{a2}/T_{a1} = \phi^3$. It is also difficult to obtain an analytical expression of the maximum relative speed of the BHMbot (v_{max}) without payloads near the resonant state. Therefore, we establish an approximate expression (power function) between v_{max} and the body length via nonlinear fitting, and the scaling is $v_{2,\ max}/v_{1,\ max} = \phi^{-0.98}$. Similarly, the analysis processes of the optimal payload mass (m_{op}) and the corresponding maximum relative speed (V_{max}) are similar, and the scaling results are $m_{op2}/m_{op1} = \phi^3$ and $V_{2,\ max}/V_{1,\ max} = \phi^{-0.86}$.

S9. Friction coefficient measurements of four flat surfaces

In this work, four flat surfaces with different levels of roughness are used to test the locomotion performance of the BHMbot. Supplementary Fig. 7a shows an experimental setup for measuring the friction coefficients between the feet of the BHMbot and four surfaces. The legs of the BHMbot are cut from a carbon fiber sheet composed of three orthogonal 50-μm-thick carbon fiber layers (Toray company) and the contact surface with the ground is the cross-section of the carbon fiber sheet. A test apparatus (9.8 g) consisting of a wood block (30 mm×30 mm×25 mm) and five beams (30 mm×2.5 mm) cut from the same carbon fiber sheet is designed, as shown in Supplementary Fig. 7b. An additional mass weighing 200 g is placed on the test apparatus to ensure sufficient contact between the apparatus and the test surfaces. A force gauge (SF-2, Aipli) is used to record the pushing force applied to the apparatus. The measurements are shown in Supplementary Table 8 and Supplementary Fig. 7c. The surface of a plastic board has the largest friction coefficient of 0.3715.

S10. COT calculation for the untethered BHMbot

The Cost of Transport (COT) is an important parameter to evaluate the energy transfer efficiency of legged locomotion[4]. Its value is defined as:

$$\mathrm{COT} = \frac{P}{mgv} \tag{27}$$

where P is the consumed power of the BHMbot, m is the total mass of the BHMbot, g is the acceleration of gravity, and v is the running speed of the BHMbot. For prototype #6, the mass m is 1.76 g and the maximum moving speed is 35 cm s^{-1} (220 Hz). We utilize two different COT values to evaluate the efficiency of the BHMbot. One is COT_M used for estimating the efficiency of the moving mechanism and P represents the output power of the electromagnetic actuators P_a. The other is COT_T used for estimating the efficiency of the whole microrobot and the P represents the output power of the battery P_b.

The COT_M can be given as:

$$\mathrm{COT_M} = \frac{P_\mathrm{a}}{mgv} = \frac{4f_\mathrm{a}\int_{-s_0}^{s_0} F_\mathrm{EM} ds}{mgv} \tag{28}$$

where f_a is the working frequency of the electromagnetic actuators (220 Hz for prototype #6); F_{EM} is the electromagnetic force of the actuators; s is the displacement of the permanent magnet relative to the coil and s_0 is the maximum displacement of the magnet during the vibration process. s_0 is confirmed by measuring the swing angle of the front leg:

$$s_0 = \frac{\theta_\mathrm{f,max}}{\lambda} \tag{29}$$

$$\lambda \approx \frac{1}{l_\mathrm{L1}} \tag{30}$$

where $\theta_{f,\,max}$ (rad) is the maximum swing angle of the front legs; λ is the transmission ratio of the transmission mechanism; l_{L1} is the length of one linkage of the transmission mechanism (0.8 mm for prototype #6) (see Supplementary Note 3). The measurement of $\theta_{f,\,max}$ is 0.26 rad and s_0 is 0.23 mm. The electromagnetic force F_{EM} is calculated via Supplementary Equation (5) and (6).

We use a galvanometer with high precision (UNI-T@ UT803) to measure the current I_c flowing the coil, which is a bipolar square wave signal. Considering the influence of the resistance of the extra wire connecting the galvanometer and the coil, we record the measurements of I_c when three segments of wire with different lengths (R_w = 0.3 Ω, 0.7 Ω, and 1.2 Ω) are connected to the circuit. The measured I_c is 0.13 A, 0.12 A, and

0.11 A. The relationship between I_c and R_w can be approximately expressed by a fitting quadratic polynomial equation:

$$I_c = 0.0056R_w^2 - 0.0306R_w + 0.1387 \tag{31}$$

When R_w drops to zero, an estimated value for I_c is 0.1387 A. We use the numerical integration method to calculate the output power of the actuators P_a and the result is 5.62 mw. Therefore,

$$\mathrm{COT_M} = \frac{P_a}{mgv} \approx 9.31 \tag{32}$$

Considering that the output voltage of the power and control circuit is 1.2 V, we can also obtain the power consumption of one electromagnetic actuator (1.2×0.1387=0.166 W). Therefore, the energy efficiency of the electromagnetic actuator is as follows.

$$\eta_a = \frac{P_a}{p_{el}} = \frac{5.62}{166 \times 2} \approx 1.7\% \tag{33}$$

where P_{el} is the output power of the power circuit.

For the total Cost of Transport $\mathrm{COT_T}$, the consumed power P_b can be expressed as:

$$P_b = V_b I_b \tag{34}$$

where V_b is the rated voltage of the battery (3.7 V), and I_b is the output current of the battery during the running movement of the BHMbot. A galvanometer (UNI-T@ UT803) is connected to the circuit of the battery to measure I_b. Similarly, we record the measurements of I_b when three segments of wire with different lengths (R_w = 0.3 Ω, 0.7 Ω, and 1.2 Ω) are connected to the circuit. The measured I_b is 0.47 A, 0.46 A, and 0.45 A (the frequencies of the two driving channels are both 220 Hz). The relationship between I_b and R_w can be approximately expressed as:

$$I_b = 0.0056R_w^2 - 0.0306R_w + 0.4787 \tag{35}$$

When R_w drops to zero, an estimated value for I_b is 0.4787 A. Therefore, the consumed power P_b is 1.77 W, and the $\mathrm{COT_T}$ is estimated based on $\mathrm{COT_T}$ = 3.7 V×0.4787 A/ (0.0017 kg×9.8 m s^{-2}×0.35 m s^{-1}) = 303.7.

Supplementary Figures 1-9

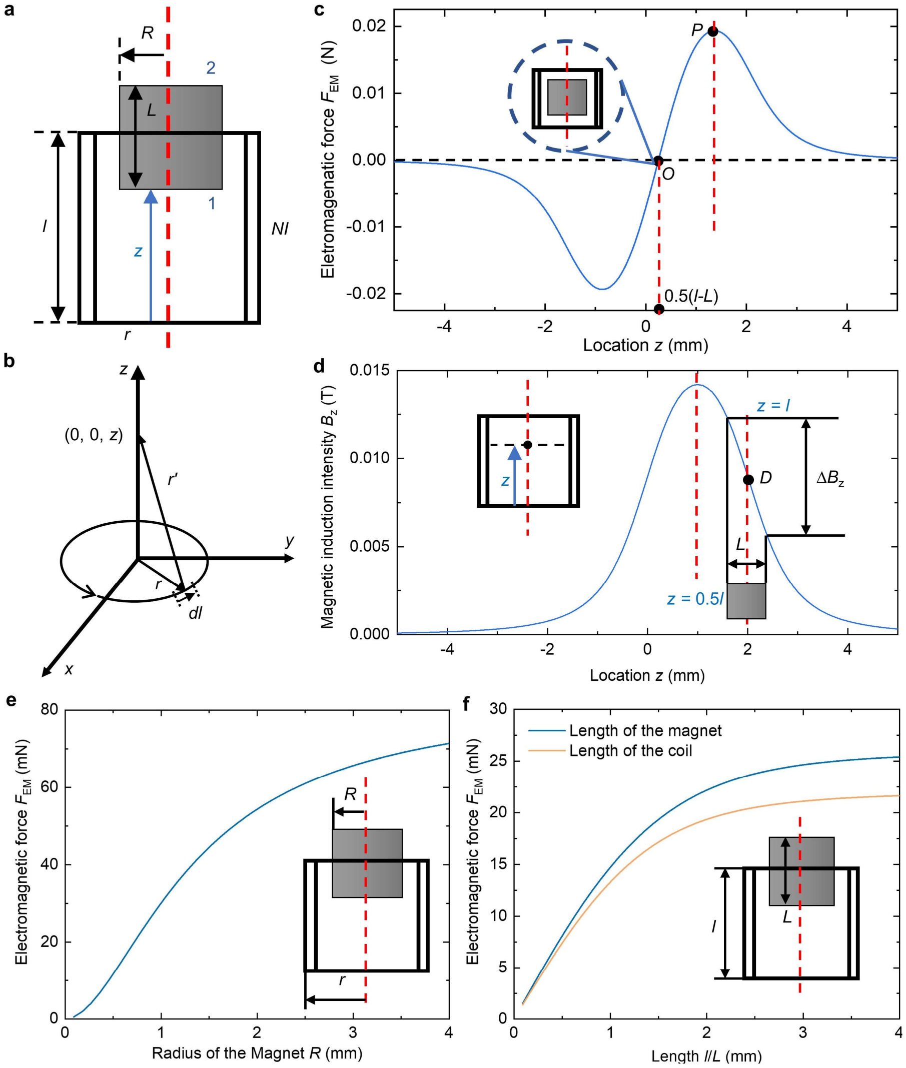

Supplementary Fig. 1. Modeling and optimization of the electromagnetic actuator. a Schematic of the electromagnetic actuator. **b** Orthogonal coordinate system for the magnetic field generated by the hollow coil. **c** Variation curve of the electromagnetic force F_{EM} versus the relative position z of the magnet in the axial direction. **d** Variation curve of the magnetic induction intensity versus the relative position z of the magnet in the axial direction. **e** Variation curve of the electromagnetic force F_{EM} versus the radius of the magnet R. **f** Variation curves of the electromagnetic force F_{EM} versus the length of the magnet L and the length of the coil l.

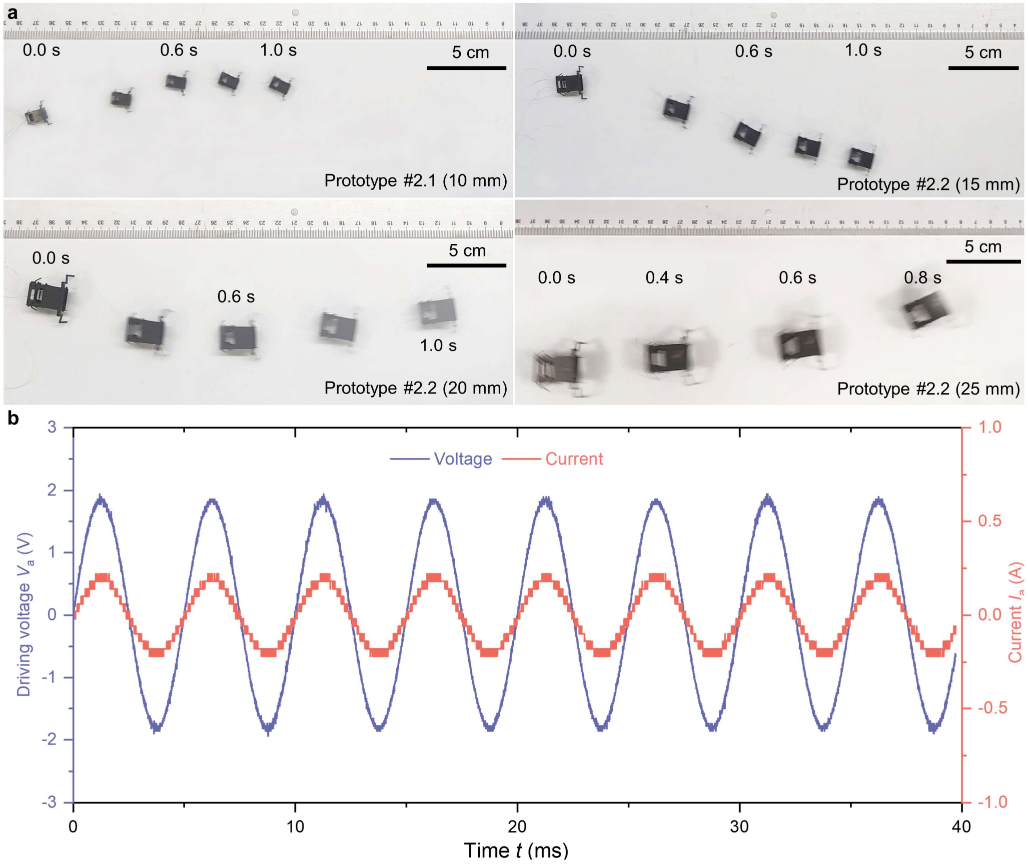

Supplementary Fig. 2. Tethered locomotion performance of the BHMbot. a Prototypes of 10, 15, 20 and 25 mm achieve measured maximum speeds of 24.1 BL s^{-1} (24.1 cm s^{-1}), 17.5 BL s^{-1} (26.2 cm s^{-1}), 14.4 BL s^{-1} (28.8 cm s^{-1}), and 11.7 BL s^{-1} (29.2 cm s^{-1}) respectively when driven by an alternating current of 0.15A. **b** Measurements of the working current and voltage signals of the 15-mm prototype (prototype #2.2) via a high-precision galvanometer when the running speed reaches its maximum (200 Hz).

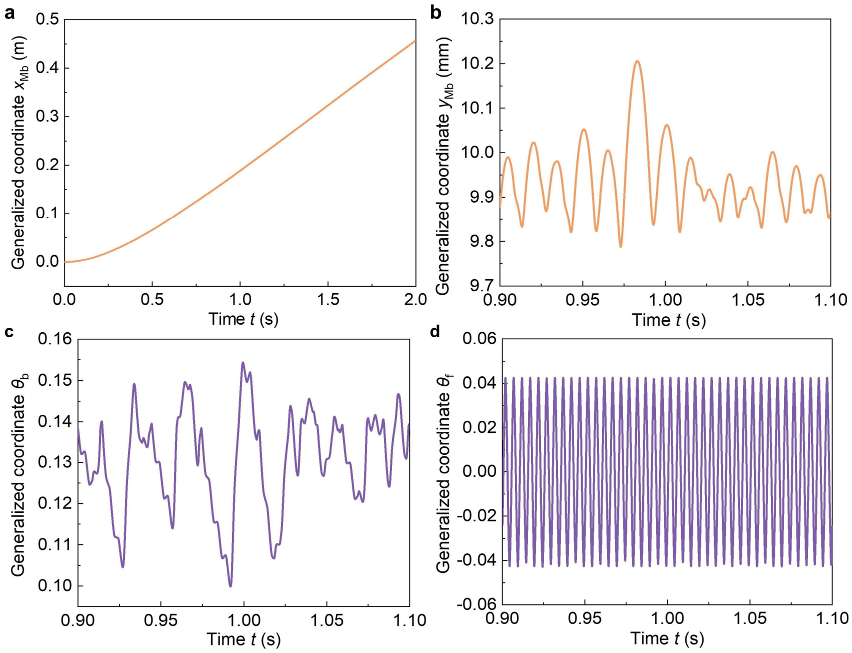

Supplementary Fig. 3. Solution results of the dynamic model. **a** Variation of the generalized coordinate x_{Mb} versus time t. **b** Variation of the generalized coordinate y_{Mb} versus time t. **c** Variation of the generalized coordinate θ_b versus time t. **d** Variation of the generalized coordinate θ_f versus time t. The driving frequency in the simulation is set to 200 Hz.

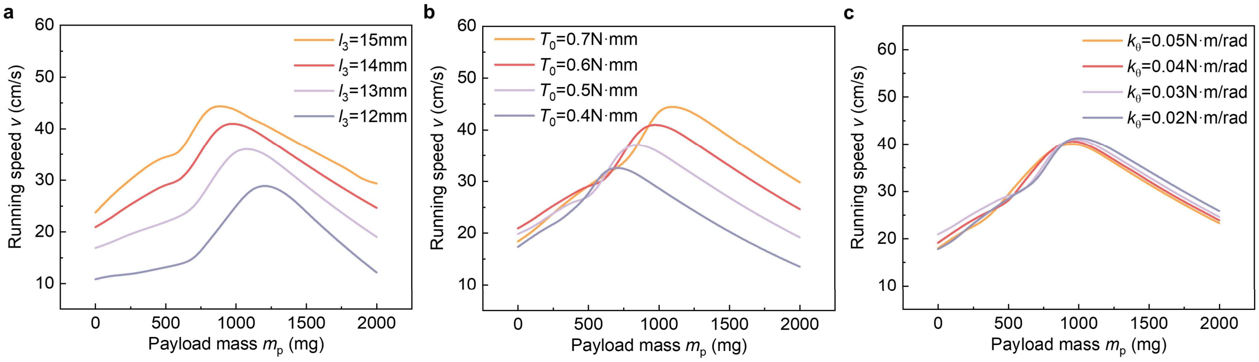

Supplementary Fig. 4. Simulation of the running speed versus payload mass. a Simulation results of the running speed versus the payload mass for the BHMbot with various relative locations of the payload l_3 (12, 13, 14, and 15 mm). **b** Simulation results of the running speed versus the payload mass for the BHMbot actuated by the active torque with various amplitudes T_0 (0.4, 0.5, 0.6, and 0.7 N·mm). **c** Simulation results of the running speed versus the payload mass for the BHMbot with various torsional stiffness k_θ (0.02, 0.03, 0.04, and 0.05 N·m/rad).

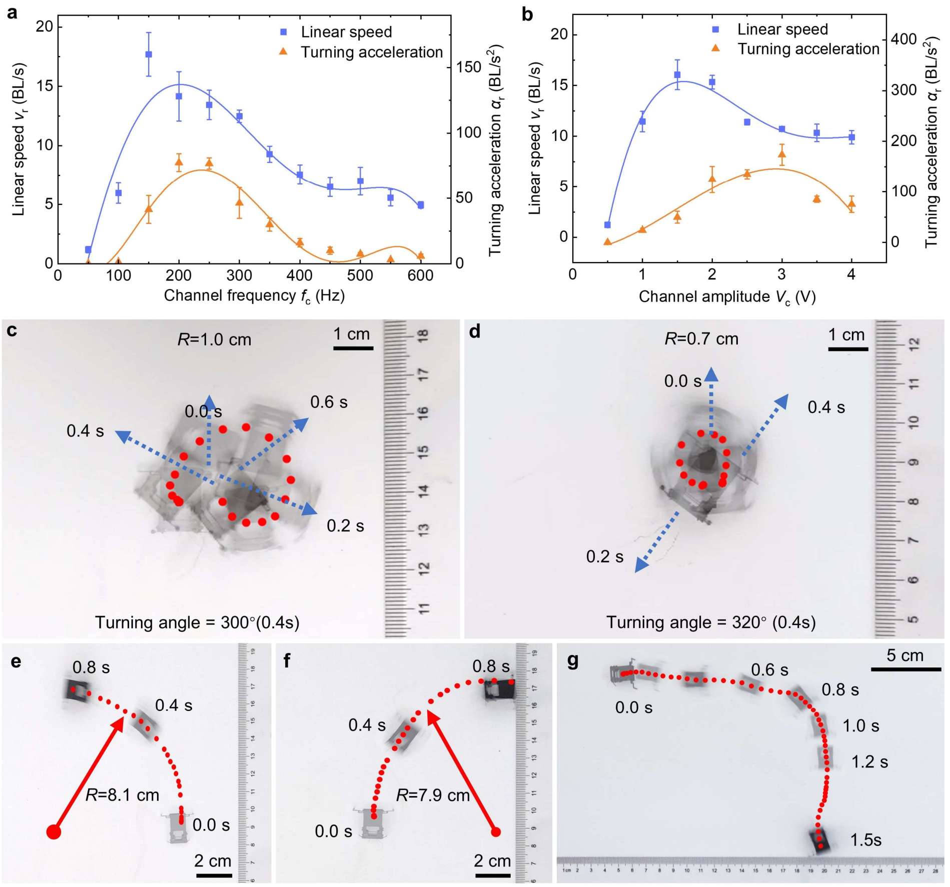

Supplementary Fig. 5. Control strategies of the tethered BHMbot. a, b Experimental results of the linear running speed (blue points) and turning centripetal acceleration (orange points) of a 15-mm-long BHMbot (prototype #2.2, 370 mg) versus the frequency and amplitude of two driving channels. All error bars represent the standard deviation of four measurements. **c** Optical photo showing the BHMbot achieving a clockwise turn of 300° in 0.4 s with a small turning radius of 1.0 cm. **d** Optical photo showing the BHMbot achieving an anticlockwise turn of 320° in 0.4 s with a small turning radius of 0.7 cm. **e** Optical photo showing the BHMbot achieving a 90-degree left turn along a curve with a radius of 8.1 cm. **f** Optical photo showing the BHMbot achieving a 90-degree right turn along a curve with a radius of 7.9 cm. **g** Optical photo showing the BHMbot achieving a controlled locomotive trajectory combined by two lines and a 90-degree turn.

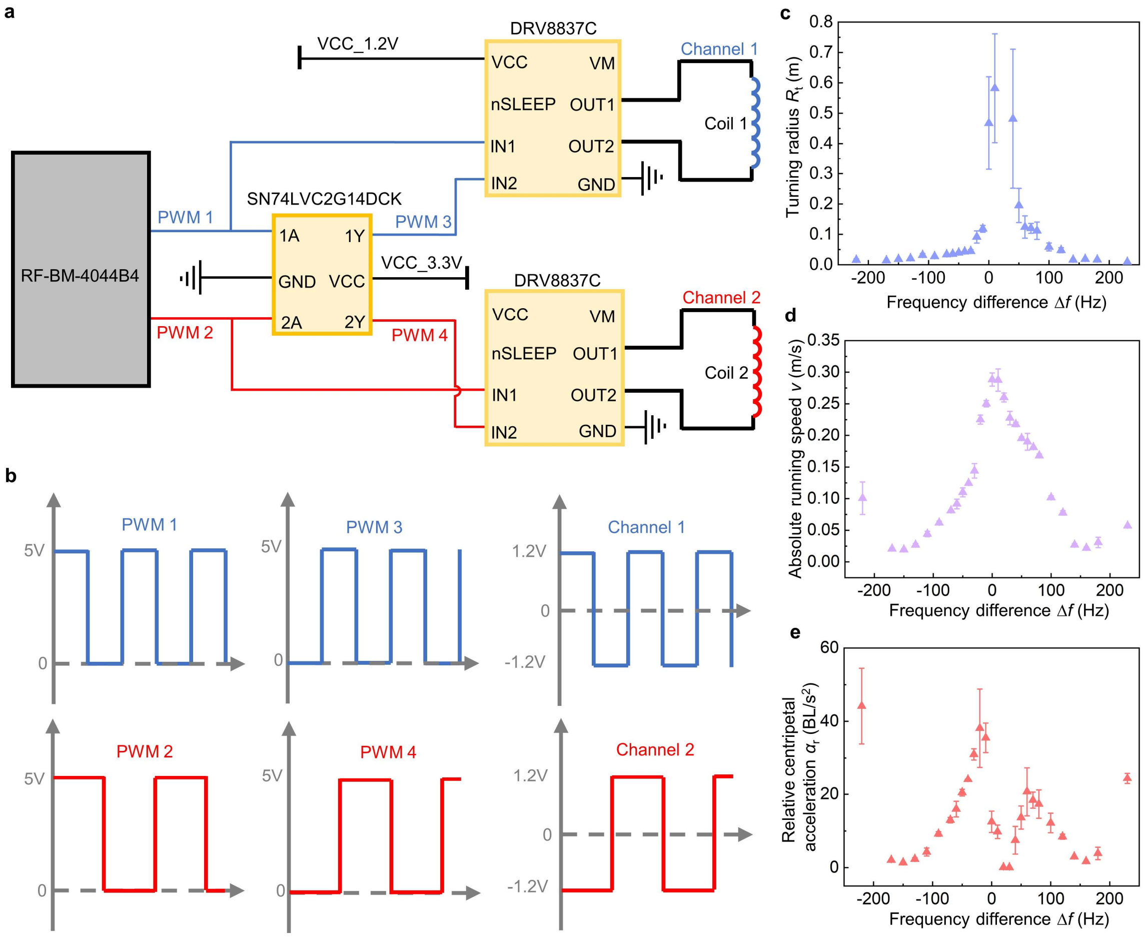

Supplementary Fig. 6. Design of the power and control circuit. a Generation of two driving channels and related electronic components. **b** Waveforms of four control PWM signals and two driving channels. **c, d, e** Turning radius, absolute linear speed, and relative centripetal acceleration with respect to the frequency difference from -230 to 230 Hz for the untethered BHMbot (prototype #5) on a plastic surface. All error bars represent the standard deviation of four measurements.

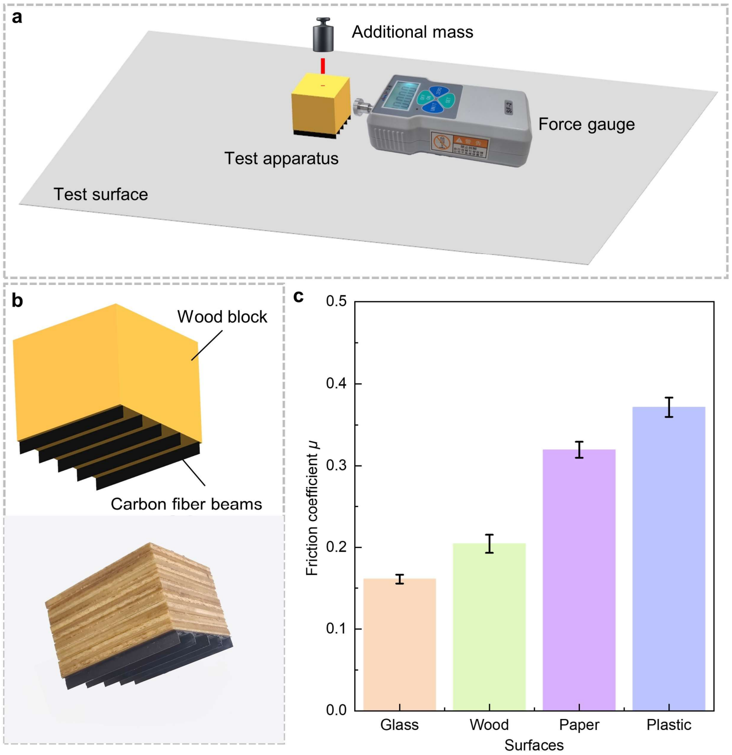

Supplementary Fig. 7. Experimental setup and measurements of the friction coefficients between the feet of the BHMbot and four different surfaces. a Experimental setup of measuring the friction coefficients. **b** Test apparatus designed to simulate the contacting area of the BHMbot with the ground. The apparatus consists of a wood block and five carbon beams. **c** Measurements of the friction coefficients of four surfaces. The error bars represent the standard deviation of four measurements.

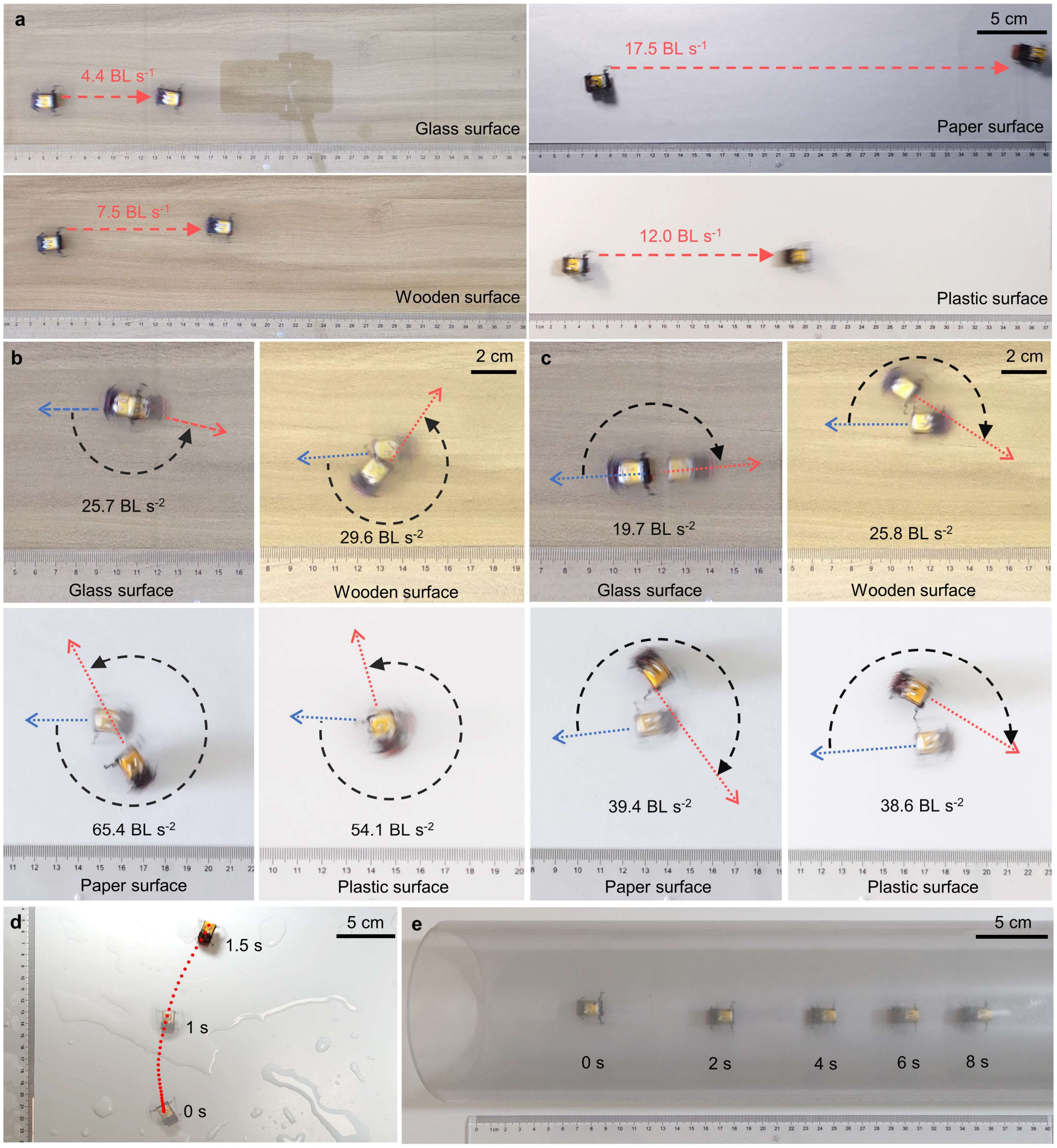

Supplementary Fig. 8. Untethered locomotion performance of the BHMbot (prototype #6). a The BHMbot runs forward on four surfaces with different levels of roughness, namely, glass, wood, paper, and plastic. The running speed on the glass, wood, paper, and plastic surfaces is 4.4 BL s^{-1}, 7.5 BL s^{-1}, 17.5 BL s^{-1}, and 12.0 BL s^{-1}, respectively. The red dotted lines represent the linear displacements of the BHMbot in 1.0 s. **b, c** Optical photos of the BHMbot achieving anticlockwise and clockwise turns on four surfaces respectively. The black dotted lines represent the angular displacements of the BHMbot in 0.5 s. **d** Optical photo of the BHMbot running through an area with puddles of water on a plastic surface. **e,** Optical photo of the BHMbot running forward in a round tube with an inner diameter of 10 cm.

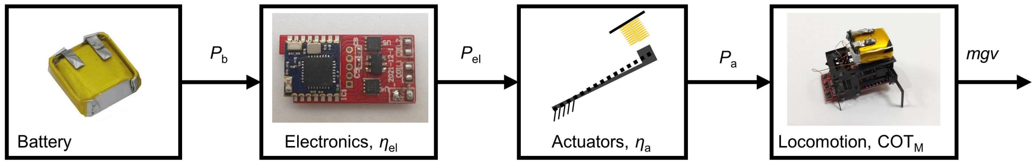

Supplementary Fig. 9. The power flow from the battery to the locomotion of the untethered BHMbot.

Supplementary Tables 1-13

Supplementary Table 1. The quantities and masses of individual components for the untethered BHMbot (prototype #6)

Description	Quantity	Mass (mg)
Support frames	--	96.94
Carbon fiber cantilevers	2	3.82
permanent magnets	2	36.3
Hollow coils	2	57.65
Fixed boards for coils	2	7.16
Metal baffles	--	38.74
Transmission mechanisms	2	10.13
Rear legs	2	2.78
Power and control electronics (including a microphone)	1	604
Lithium battery	1	780
Wires and glue	--	≈ 5
Total		≈ 1760

Supplementary Table 2. Comparison of the estimate and theoretical values of the optimal relative distance between the magnet and the coil for the actuator

	Relative distance of the magnet and the coil z (mm)			
	0.5	0.75	1	1.25
Estimate (l-L/2)	1.75	1.5	1.25	1
Theoretical value (mN)	1.79	1.57	1.37	1.23
Relative error	8%	7%	8%	11.5%

Supplementary Table 3. Optimized geometric parameters of the actuator and the revised values of these parameters considering the fabrication limitations

Parameters	Optimized	Revised	Relative error
Radius of the hollow coil r	1.225825	1.25	2%
Length of the hollow coil l	2.310518	2.0	13.4%
Radius of the permanent magnet R	1.075825	1	7%
Length of the permanent magnet L	1.810518	1.5	17.1%

Supplementary Table 4. The size of the components of four tethered prototypes with different body lengths (prototype #2.1-2.4, with lengths of 10 mm, 15 mm, 20 mm, and 25 mm)

Prototype	Components	Parameter	Values
Prototype #2.1 (10 mm)	Hollow coil	diameter	2.0 mm
		length	1.4 mm
	Magnet	diameter	1.5 mm
		length	1.0 mm
	Cantilever	length	8 mm
		width	0.8 mm
	Upper support frame	length	10 mm
		width	8 mm
	Front support frame	length	8 mm
		width	4 mm
	Hinge of the transmission	thickness	15 μm
Prototype #2.2 (15 mm)	Hollow coil	diameter	2.5 mm
		length	2.0 mm
	Magnet	diameter	2.0 mm
		length	1.5 mm
	Cantilever	length	12 mm
		width	1.2 mm
	Upper support frame	length	15 mm
		width	10 mm
	Front support frame	length	10 mm
		width	5 mm
	Hinge of the transmission	thickness	20 μm
Prototype #2.3 (20 mm)	Hollow coil	diameter	3.6 mm
		length	2.7 mm
	Magnet	diameter	2.5 mm
		length	2.0 mm
	Cantilever	length	16 mm
		width	1.6 mm
	Upper support frame	length	20 mm
		width	13.3 mm
	Front support frame	length	13.3 mm
		width	6.7 mm
	Hinge of the transmission	thickness	25 μm
Prototype #2.4 (25 mm)	Hollow coil	diameter	4.2 mm
		length	3.4 mm
	Magnet	diameter	3.5 mm
		length	2.5 mm
	Cantilever	length	20 mm
		width	2.0 mm
	Upper support frame	length	25 mm
		width	16.7 mm
	Front support frame	length	16.7 mm
		width	8.3 mm
	Hinge of the transmission	thickness	30 μm

Supplementary Table 5. The running speed of the tethered BHMbot versus the payload mass (prototype #2.2)

Payload mass (mg)	Maximum Running speed (cm s^{-1})	Resonance frequency (Hz)
0	26.4 ± 1.9	265 ± 5
200	26.9 ± 0.2	265 ± 5
400	27.6 ± 0.9	260 ± 5
600	28.7 ± 3.1	255 ± 5
800	32.6 ± 1.1	250 ± 5
1000	40.9 ± 4.8	245 ± 5
1200	43.8 ± 0.9	245 ± 5
1400	38.4 ± 2.6	240 ± 5
1600	29.4 ± 1.3	240 ± 5
1800	24.6 ± 1.1	235 ± 5
2000	21.8 ± 0.5	230 ± 5

Supplementary Table 6. Parameters in the dynamic simulations (for 15-mm prototype)

Parameters	Description	Values in simulation
m_{f}	Mass of the front leg	40 mg
m_{b}	Mass of the rigid body	360 mg
m_{p}	Mass of the payload	0-10 g
J_{f}	Moment of inertia of the front leg M_{f}	3.0×10^{-10} kg·m^2
J_{b}	Moment of inertia of the rigid body M_{b}	3.3×10^{-8} kg·m^2
ρ_{air}	Density of air	1.293 kg m^{-3}
BL	Length of the rigid body	15 mm
θ_0	Initial tilt angle of the body	0-15°
l_{r}	Length of the rear leg	3-10 mm
C_{D}	Air damping coefficient	3.4
k	Elastic coefficient of the spring-damper model between the feet and the ground	200
d	Damping coefficient of the spring-damper model between the feet and the ground	0.2
μ	Coefficient of the friction between the feet and the ground	0.2

Supplementary Table 7. Scaling effects of the tethered BHMbot

Parameters	Model approach scale
Body length BL_2/BL_1	ϕ
Total mass m_2/m_1	ϕ^3
Torsional stiffness $k_{\theta 2}/k_{\theta 1}$	ϕ^3
Resonant frequency f_{r2}/f_{r1}	ϕ^{-1}
Electromagnetic force of the actuator F_{EM2}/F_{EM1}	ϕ^2
Torque of the actuator T_{a2}/T_{a1}	ϕ^3
Maximum relative speed without payloads $v_{2, max}/v_{1, max}$	$\phi^{-0.98}$
Optimal payload mass m_{op2}/m_{op1}	ϕ^3
Maximum relative speed with optimal payloads $V_{2, max}/V_{1, max}$	$\phi^{-0.86}$

Supplementary Table 8. Measurements of the friction coefficients of four substrates

Substrate	Friction force (N)		Friction coefficient	
	Average	Standard deviation	Average	Standard deviation
glass	0.3222	0.0109	0.1611	0.0055
wood	0.4087	0.0223	0.20435	0.0111
paper	0.6392	0.0195	0.3196	0.0098
plastic	0.7430	0.0236	0.3715	0.0118

Supplementary Table 9. The detailed performance parameters of turning tests on four substrates of the untethered BHMbot (prototype #6)

Substrate		Absolute moving speed (cm s^{-1})	Turning angular speed (rad s^{-1})	Turning radius (cm)	Relative centripetal acceleration (BL s^{-2})	Frequency of driving channels (Hz)
Glass	clockwise	5.85	6.50	0.90	19.7	249, 0
	anticlockwise	5.99	8.56	0.70	25.7	0, 205
Wood	clockwise	7.1	7.24	0.98	25.7	205, 0
	anticlockwise	7.21	8.19	0.88	29.5	0, 220
Paper	clockwise	10.91	9.92	1.10	39.4	219, 0
	anticlockwise	13.53	9.66	1.40	65.4	0, 230
Plastic	clockwise	9.82	7.86	1.25	38.5	217, 0
	anticlockwise	9.76	11.09	0.88	54.1	0, 227

Supplementary Table 10. Data of relative speed with respect to the mass of some animals and untethered legged robots

Species	Body mass (g)	Relative speed (BL s^{-1})	Reference
Mammals			
Elephas maximus	4000000	1.18	5
Bison	700000	5.158725	6
Ovis ammon	114000	10.4130375	6
Rangifer tarandus	160000	9.72	6
Lycaon pictus	22000	18.3526	6
Urocyon cinereoargenteus	3700	29.6	7
Uromys caudimaculatus	1180	16.6	7
Equus zebra	300000	7.35	7
Acinonyx jubatus	58800	22.67	7
Insects			
Cockroach (*Nauphoeta cinerea*)	0.45	13	8
Cockroach (*Periplaneta americana*)	0.83	50	9
Ant (*Leptogenys schwabi*)	0.00896	1.36	10
Ant (*Formica fusca L.*)	0.0047	2.6	11
Spider (*Eremobates marathoni*)	3.49	9.9	12
Spider (*Dolomedes plantarius*)	1.5	37.5	12
Beetle (*Onymacris plana*)	0.73	50	13
Beetle (*Cicindela eburneola*)	0.05	171	14
Mite (*Paratarsotomus macropalpis*)	0.027	192.4	15
Untethered artificial robots			
HAMR-F	2.8	3.8	16
Soft robot by Liu et al	2.19	2.5	17
PZT robot by Cuenca et al	7.068	0.56	18
SEMR UL	0.71	1.2	19
Soft robot by Lin et al	1.9	1.2	20
Electrostatically driven robot by Zhu et al	0.0464	1.18	21
RoBeetle	0.088	0.05	22
DEAnsect	1	0.3	23
S^2worm	4.34	6.7	24
RoACH	2.4	1	25
Kilobot	36	0.3	26
µTugs	12	0.4	27
Colias	28	8.75	28
R-one	230	3	29
Inchy	20	11.8	30
PAW	15700	2.4	31
Takken 2	4300	3.16	32
Cheetah	850	1.1	33
Puppy 2	273	3.5	34
MilliMobile	1.1	0.55	35
This work			
BHMbot (prototype #6)	1.7	17.5	--

Supplementary Table 11. Data of relative centripetal acceleration with respect to body length of some animals and legged robots

Species	Body length (m)	Relative centripetal acceleration (BL s^{-2})	Reference
Mammal			
Cheetah	1.05	12.34	[36]
Impala	1.02	10.91	[36]
Lion	1.45	5.20	[36]
Zebra	1.46	4.46	[36]
Horse	~1.55	5.16	[37]
Rats	~0.24	26.42	[38]
Human	~1.75	5.11	[39]
Insects			
Whirligig beetles	0.0124	842.91	[40]
Honeybee	0.016	5.27	[41]
Spider	0.005	2191.02	[42]
Cockroach	0.044	14.32	[43]
Ant	0.0034	7.92	[44]
Untethered artificial robots			
DEAnsect	0.04	0.04	[23]
S^2worm	0.041	0.20	[24]
Hexapod robot with six wheels	0.12	1.11	[45]
PISCES	0.09	2.51	[46]
TAYLRoACH	0.1	25.6	[47]
Resilient soft robot	0.65	2.56e-5	[48]
Waalbot	0.13	0.32	[49]
This work			
BHMbot (prototype #6)	0.02	65.4	--

Supplementary Table 12. Detailed dimensions of six 15-mm prototypes used in this paper

	Prototype #1and #2.2	Prototype #3	Prototype #4	Prototype #5	Prototype #6
Description	Not optimized	optimized	Optimized	Optimized	Optimized
Integrate with power and control units	No	No	No	Yes	Yes
Integrate with a sensor unit	No	No	No	No	Yes
Total length	15 mm	15 mm	15 mm	20 mm	20 mm
Total mass	370 mg	380 mg	380 mg	1700 mg	1760 mg
Cantilever width	1.2 mm	1.4 mm	1.5 mm	1.5 mm	1.5 mm
Relative distance between the coil and the magnet	1.0 mm	1.5 mm	1.5 mm	1.5 mm	1.5 mm
Initial body tilt angle	10°	9°	8°	8°	8°
Length of rear legs	8 mm	7 mm	7 mm	7 mm	7 mm

Supplementary Table 13. Components of the power and control circuit board

Description	Model	Quantity	Weight (mg)
Microcontroller	RF-BM-4044B4	1	193.1
Voltage regulator (3.3V)	SPX3819M5	1	14.8
Voltage regulator (1.2V)	TLV75709PDBVR	1	15.2
Schmitt-Trigger inverter	SN74LVC2G14DCK	1	16.4
H-Bride driver	DRV8837C	2	19.6
Sensor (a microphone)	ICS-40181	1	60.0
Capacitor	0402; 0603; 0805	11	111.0
PCB	L: 15mm; W:10mm; (143 mm^2)	1	120.0
Solder			~50
Total weight			~600

Supplementary References

1 Song, C.-W. & Lee, S.-Y. Design of a Solenoid Actuator with a Magnetic Plunger for Miniaturized Segment Robots. *Applied Sciences* **5**, 595-607 (2015).

2 Wood, R. J. Liftoff of a 60mg flapping-wing MAV. in *2007 IEEE/RSJ International Conference on Intelligent Robots and Systems.* 1889-1894.

3 Wood, R. J. Design, fabrication, and analysis of a 3DOF, 3cm flapping-wing MAV. in *2007 IEEE/RSJ International Conference on Intelligent Robots and Systems.* 1576-1581.

4 Kim, S. & Wensing, P. M. Design of dynamic legged robots. *Foundations and Trends® in Robotics* **5**, 117-190, (2017).

5 Alexander, R. M. Allometry of the limbs of antelopes (Bovidae). *J. Zool.* **183**, 125-146, (1977).

6 CHRISTIANSEN, P. Locomotion in terrestrial mammals: the influence of body mass, limb length and bone proportions on speed. *Zool. J. Linn. Soc.* **136**, 685-714, (2002).

7 Alexander, R. M., Langman, V. & Jayes, A. Fast locomotion of some African ungulates. *J. Zool.* **183**, 291-300, (1977).

8 Weihmann, T., Brun, P.-G. & Pycroft, E. Speed dependent phase shifts and gait changes in cockroaches running on substrates of different slipperiness. *Front. Zool.* **14**, 54, (2017).

9 Full, R. J. & Tu, M. S. Mechanics of a rapid running insect: two-, four-and six-legged locomotion. *J. Exp. Biol.* **156**, 215-231, (1991).

10 Duncan, F. & Crewe, R. A comparison of the energetics of foraging of three species of Leptogenys (Hymenoptera, Formicidae). *Physiol. Entomol.* **18**, 372-378, (1993).

11 Jensen, T. F. & Holm-Jensen, I. Energetic cost of running in workers of three ant species,Formica fusca L.,Formica rufa L., andCamponotus herculeanus L. (Hymenoptera, Formicidae). *Journal of comparative physiology* **137**, 151-156, (1980).

12 Punzo, F. The Effects of Reproductive Status on Sprint Speed in the Solifuge, Eremobates marathoni (Solifugae, Eremobatidae). *The Journal of Arachnology* **26**, 113-116, (1998).

13 Bartholomew, G. A., Lighton, J. R. B. & Louw, G. N. Energetics of locomotion and patterns of respiration in tenebrionid beetles from the Namib Desert. *Journal of Comparative Physiology B* **155**, 155-162, (1985).

14 Kamoun, S. & Hogenhout, S. A. Flightlessness and Rapid Terrestrial Locomotion in Tiger Beetles of the Cicindela L. Subgenus Rivacindela van Nidek from Saline Habitats of Australia (Coleoptera: Cicindelidae). *The Coleopterists Bulletin* **50**, 221-230, (1996).

15 Rubin, S., Young, M. H.-Y., Wright, J. C., Whitaker, D. L. & Ahn, A. N. Exceptional running and turning performance in a mite. *J. Exp. Biol.* **219**, 676-685, (2016).

16 Goldberg, B. *et al.* Power and Control Autonomy for High-Speed Locomotion With an Insect-Scale Legged Robot. *IEEE Rob. Autom. Lett.* **3**, 987-993, (2018).

17 Miao, Z. *et al.* Power Autonomy and Agility Control of an Untethered Insect-Scale Soft Robot. *Soft Rob.* **10**, 749-759, (2023).

18 Robles-Cuenca, D., Ramírez-Palma, M. R., Ruiz-Díez, V., Hernando-García, J. & Sánchez-Rojas, J. L. Miniature Autonomous Robot Based on Legged In-Plane Piezoelectric Resonators with Onboard Power and Control. *Micromachines* **13** (2022).

19 Mao, G. *et al.* Ultrafast small-scale soft electromagnetic robots. *Nat. Commun.* **13**, 4456, (2022).

20 Liang, J. *et al.* Electrostatic footpads enable agile insect-scale soft robots with trajectory control. *Sci. Rob.* **6**, eabe7906, (2021).

21 Zhu, Y. *et al.* A 5-mm Untethered Crawling Robot via Self-Excited Electrostatic Vibration. *IEEE Trans. Rob.* **38**, 719-730, (2022).

22 Yang, X., Chang, L. & Pérez-Arancibia Néstor, O. An 88-milligram insect-scale autonomous crawling robot driven by a catalytic artificial muscle. *Sci. Rob.* **5**, eaba0015, (2020).

23 Ji, X. *et al.* An autonomous untethered fast soft robotic insect driven by low-voltage dielectric elastomer actuators. *Sci.*

Rob. **4**, eaaz6451, (2019).
24 Liu, Y. *et al.* S^{2}worm: A Fast-Moving Untethered Insect-Scale Robot With 2-DoF Transmission Mechanism. *IEEE Rob. Autom. Lett.* **7**, 6758-6765, (2022).
25 Hoover, A. M., Steltz, E. & Fearing, R. S. RoACH: An autonomous 2.4g crawling hexapod robot. in *2008 IEEE/RSJ International Conference on Intelligent Robots and Systems.* 26-33.
26 Rubenstein, M., Ahler, C. & Nagpal, R. Kilobot: A low cost scalable robot system for collective behaviors. in *2012 IEEE International Conference on Robotics and Automation.* 3293-3298.
27 Christensen, D. L., Hawkes, E. W., Suresh, S. A., Ladenheim, K. & Cutkosky, M. R. µTugs: Enabling microrobots to deliver macro forces with controllable adhesives. in *2015 IEEE International Conference on Robotics and Automation (ICRA).* 4048-4055.
28 Arvin, F., Murray, J., Zhang, C. & Yue, S. Colias: An autonomous micro robot for swarm robotic applications. *Int. J. Adv. Rob. Syst.* **11**, 113, (2014).
29 McLurkin, J. *et al.* in *Distributed Autonomous Robotic Systems: The 10th International Symposium* (eds Alcherio Martinoli *et al.*) 597-609 (Springer Berlin Heidelberg, 2013).
30 Caprari, G. *Autonomous micro-robots: Applications and limitations*, Verlag nicht ermittelbar, (2003).
31 Smith, J. A., Poulakakis, I., Trentini, M. & Sharf, I. Bounding with active wheels and liftoff angle velocity adjustment. *The International Journal of Robotics Research* **29**, 414-427, (2010).
32 Kimura, H., Fukuoka, Y. & Cohen, A. H. Adaptive Dynamic Walking of a Quadruped Robot on Natural Ground Based on Biological Concepts. *The International Journal of Robotics Research* **26**, 475-490, (2007).
33 Spröwitz, A. *et al.* Towards dynamic trot gait locomotion: Design, control, and experiments with Cheetah-cub, a compliant quadruped robot. *The International Journal of Robotics Research* **32**, 932-950, (2013).
34 Iida, F., Gomez, G. & Pfeifer, R. Exploiting body dynamics for controlling a running quadruped robot. in *ICAR '05. Proceedings., 12th International Conference on Advanced Robotics, 2005.* 229-235.
35 Johnson, K. *et al.* in *Proceedings of the 29th Annual International Conference on Mobile Computing and Networking* Article 90 (Association for Computing Machinery, 2023).
36 Wilson, A. M. *et al.* Biomechanics of predator–prey arms race in lion, zebra, cheetah and impala. *Nature* **554**, 183-188, (2018).
37 Tan, H. & Wilson, A. M. Grip and limb force limits to turning performance in competition horses. *Proceedings of the Royal Society B: Biological Sciences* **278**, 2105-2111, (2011).
38 Walter, R. M. Kinematics of 90 running turns in wild mice. *J. Exp. Biol.* **206**, 1739-1749, (2003).
39 Chang, Y.-H. & Kram, R. Limitations to maximum running speed on flat curves. *J. Exp. Biol.* **210**, 971-982, (2007).
40 Fish, F. E. & Nicastro, A. J. Aquatic turning performance by the whirligig beetle: constraints on maneuverability by a rigid biological system. *J. Exp. Biol.* **206**, 1649-1656, (2003).
41 Zolotov, V., Frantsevich, L. & Falk, E.-M. Kinematik der phototaktischen Drehung bei der HonigbieneApis mellifera L. *Journal of comparative physiology* **97**, 339-353, (1975).
42 Zeng, Y. & Crews, S. Biomechanics of omnidirectional strikes in flat spiders. *J. Exp. Biol.* **221**, jeb166512, (2018).
43 Jindrich, D. L. & Full, R. J. Many-legged maneuverability: dynamics of turning in hexapods. *J. Exp. Biol.* **202**, 1603-1623, (1999).
44 Pearce-Duvet, J. M. C., Elemans, C. P. H. & Feener, D. H., Jr. Walking the line: search behavior and foraging success in ant species. *Behav. Ecol.* **22**, 501-509, (2011).
45 Zarrouk, D. & Fearing, R. S. Controlled In-Plane Locomotion of a Hexapod Using a Single Actuator. *IEEE Trans. Rob.* **31**, 157-167, (2015).
46 Hariri, H. H., Soh, G. S., Foong, S. & Wood, K. L. A Highly Manoeuvrable and Untethered Under-Actuated Legged Piezoelectric Miniature Robot. in *ASME 2019 International Design Engineering Technical Conferences and Computers and Information in Engineering Conference.*

47 Kohut, N. J., Pullin, A. O., Haldane, D. W., Zarrouk, D. & Fearing, R. S. Precise dynamic turning of a 10 cm legged robot on a low friction surface using a tail. in *2013 IEEE International Conference on Robotics and Automation.* 3299-3306.

48 TolleyMichael, T., ShepherdRobert, F., GallowayKevin, C., WoodRobert, J. & WhitesidesGeorge, M. A resilient, untethered soft robot. *Soft Rob.*, (2014).

49 Murphy, M. P. & Sitti, M. Waalbot: An Agile Small-Scale Wall-Climbing Robot Utilizing Dry Elastomer Adhesives. *IEEE/ASME Trans. Mechatron.* **12**, 330-338, (2007).
