# Supplementary material for: A wireless controlled robotic insect with ultrafast untethered running speeds
Source: Nat Commun. 2024 May 8;15:3815. doi: 10.1038/s41467-024-47812-5 (PMC11078929; doi:10.1038/s41467-024-47812-5)
Supplement: Supplementary file 3 — Description of Additional Supplementary Files [file 41467_2024_47812_MOESM3_ESM.pdf]

Description of Additional Supplementary Files

Legends and captions of Supplementary Movies 1 to 16

Supplementary Movie 1. Swing motions of the front legs of the tethered BHMbot (prototype #1)

This high-speed movie shows a tethered BHMbot (prototype #1) swinging its front legs at 200 Hz. In part 1, the BHMbot is driven by two in-phase square signals (0.15 A, 200 Hz). In part 2, the BHMbot is driven by two out-of-phase square signals (0.15 A, 200 Hz).

Supplementary Movie 2. Bouncing movements of the tethered BHMbot (prototype #1) with a payload of 0 mg and 1200 mg

This high-speed movie shows the motion posture of the tethered BHMbots (prototype #1) when carrying a payload of 0 mg and 1200 mg. In part 1, prototype #1 with a payload of 0 mg shows a long aerial phase (with a duty cycle > 60%) and a large bounce length (0.2 body length). In part 2, prototype #1 with a payload of 1200 mg shows a shorter aerial phase (with a duty cycle < 25%) and a smaller bounce length (0.02 body length).

Supplementary Movie 3. Locomotion performance of the scaling BHMbot (prototype #2.1-2.4) near the resonant state

This movie shows the locomotion performance of four prototypes with different body lengths ranging from 10 to 25 mm at an interval of 5 mm (prototype #2.1-2.4) near the resonant state. Part 1 shows the prototypes of 10, 15, 20 and 25 mm achieve measured maximum speeds of 24.1 BL/s (24.1 cm/s), 17.5 BL/s (26.2 cm/s), 14.4 BL/s (28.8 cm/s), and 11.7 BL/s (29.2 cm/s) respectively when carrying a payload of 0 mg. Part 2 shows the prototypes of 10, 15, 20 and 25 mm achieve measured maximum speeds of 18.2 BL/s (18.2 cm/s), 29.2 BL/s (43.8 cm/s), 21.4 BL/s (42.8 cm/s), and 15.6 BL/s (39.0 cm/s) near the resonant state when carrying a payload of 200mg, 1200, 2400 and 4800 mg respectively.

Supplementary Movie 4. Locomotion performance comparison of the initial BHMbot (prototype

#2.2) and two optimized BHMbot (prototype #3 and #4)

This movie shows the comparison of the locomotion performance of three prototypes (prototype #2.2, #3 and #4). Part 1 shows prototype #2.2 and prototype #3 achieve a maximum speed of 17.5 BL/s and 33.3 BL/s respectively when carrying a payload of 0 mg. Part 2 shows prototype #2.2 and prototype #4 achieve a maximum speed of 14.5 BL/s and 25 BL/s respectively when carrying a payload of 2000 mg (a hexagonal nut, 5.26 times its mass).

Supplementary Movie 5. Three basic controlled movements of the tethered BHMbot (prototype #2.2)

This movie illustrates the control strategy of the BHMbot (prototype #2.2) via three basic movements. Part 1 shows the BHMbot achieves a clockwise turn when only the left front leg is actuated. Part 2 shows the BHMbot achieves an anticlockwise turn when only the right front leg is actuated. Part 3 shows the BHMbot achieves a straight movement when two front legs are driven by two in-phase signals respectively.

Supplementary Movie 6. Designed trajectories demonstration of the tethered BHMbot (prototype #2.2) on a plastic board

This movie shows the trajectory control of the BHMbot (prototype #2.2) driven by programmed driving channels. Part 1 shows the BHMbot achieves a 300° clockwise turn with a radius of 1.0 cm in 0.4 s. Part 2 shows the BHMbot achieves a 320° anticlockwise turn with a radius of 0.7 cm in 0.4 s. Part 3 shows the BHMbot achieves a 90° right turn with a radius of 8.1 cm in 0.8 s. Part 4 shows the BHMbot achieves a left turn with a radius of 7.9 cm in 0.8 s. Part 5 shows the BHMbot achieves a continuous movement consisting of two linear paths and a 90° turn in 1.5 s.

Supplementary Movie 7. Designed trajectories demonstration of the untethered BHMbot (prototype #5) on a plastic board

This movie shows the BHMbot (prototype #5) carrying a lithium battery and a power and control circuit runs along designed trajectories under programmed commands from a host computer. Part 1 demonstrates a circular trajectory with a diameter of 10.5 cm and part 2 demonstrates a rectangle trajectory with a length of 11.5 cm and a width of 14 cm.

Supplementary Movie 8. Capitals (BUAA) trajectories demonstration of the untethered BHMbot (prototype #5) on a plastic board

This movie shows the BHMbot (prototype #5) running along complicated trajectories under programmed commands from a host computer, including four capitals “BUAA”.

Supplementary Movie 9. Obstacles avoidance demonstration of the untethered BHMbot (prototype #5) via remote control

This movie shows that the BHMbot (prototype #5) runs through a 40 cm-by-60 cm area scattered with stones and leaves under remote control from a smartphone in 70 s. Part 1 shows the top view of the whole moving process. Part 2 shows the side view of the process in which the BHMbot runs through a narrow tunnel built of stones.

Supplementary Movie 10. Straight running test of the optimized untethered BHMbot (prototype #6) on four flat surfaces

This movie shows the straight running movements of the optimized untethered BHMbot (prototype #6) on four flat surfaces with different levels of roughness, including a glass sheet, a wood desktop, a printed paper, and a plastic board. The BHMbot achieves a maximum speed of 17.5 BL/s on the printed paper.

Supplementary Movie 11. Turning test of the optimized untethered BHMbot (prototype #6) on four flat surfaces

This movie shows the clockwise and anticlockwise turns of the optimized untethered BHMbot (prototype #6) on four different surfaces, including a glass sheet, a wood desktop, a printed paper, and a plastic board. The BHMbot achieves a maximum turning centripetal acceleration of 39.4 BL/s^2 (clockwise) and 65.4 BL/s^2 (anticlockwise) on the printed paper.

Supplementary Movie 12. Locomotion demonstration of the untethered BHMbot (prototype #6) on a flat surface with puddles and a curved surface

This movie demonstrates the locomotion of the BHMbot on two more complex surfaces. Part 1 shows the BHMbot (prototype #6) runs on a plastic board with puddles of water with a maximum speed of 10 BL/s. Part 2 shows the BHMbot (prototype #6) runs in a plastic round tube with a diameter of 10 cm. The maximum speed of the BHMbot is 2.5 BL/s.

Supplementary Movie 13. Maximum duration test of the untethered BHMbot (prototype #6) on a flat plastic board (3 min)

This movie demonstrates that the maximum duration of prototype #6 can reach 3 minutes when powered by a lithium battery of 50 mAh.

Supplementary Movie 14. Sound detection demonstration of the untethered BHMbot (prototype #6 with a microphone sensor integrated) via remote control

This movie shows the sound detection demonstration of the BHMbot (prototype #6). Part 1 shows the BHMbot runs to a collapsed house built of blocks and receives SOS signals from a Bluetooth speaker in the house. Then the BHMbot returns to the start point. Part 2 shows the waveform of the received SOS signals, which is displayed on the smartphone.

Supplementary Movie 15. Demonstration of the untethered BHMbot (prototype #6) running inside two aero engines via remote control

This movie shows the untethered BHMbot (prototype #6) running inside two aero engines. In part 1, the main picture shows the BHMbot runs through a passage between two stator blades and returns to the start point from the front view. The minor picture at the bottom right shows the process from the back view. In part 2, the BHMbot runs quickly with a maximum speed of 4.5 BL/s in the tail cone of a turbojet engine.

Supplementary Movie 16. Transportation demonstration of the untethered BHMbot (prototype #6) via a quadrotor

This movie shows the BHMbot (prototype #6) collaborates with a quadrotor to execute tasks on a desktop. Part 1 shows a distant view that the BHMbot is transported to a desktop by the quadrotor from the ground. Part 2 shows a closer view that the quadrotor lands on the desktop and the BHMbot runs out of the nacelle. Then the BHMbot circles back to the nacelle after passing a stone and the quadrotor flies off the desktop with the BHMbot. Part 3 shows a distant view that the quadrotor flies off the desktop with the BHMbot.
